# Using a Theoretical Framework in a Qualitative Metasummary About Fatigue Adaptation After Stroke

**DOI:** 10.1093/geroni/igab046.649

**Published:** 2021-12-17

**Authors:** Chiao-Hsin Teng, Ruth Anderson, Leslie Davis

**Affiliations:** 1 UNC Chapel Hill School of Nursing, Chapel Hill, North Carolina, United States; 2 University of North Carolina at Chapel Hill, UNC Chapel Hill, North Carolina, United States; 3 The University of North Carolina at Chapel Hill, Chapel Hill, North Carolina, United States

## Abstract

We describe how we used a theoretical framework, Adaptive Leadership Framework for Chronic Illness (ALFCI), to complete a qualitative metasummary in a scoping review of 26 articles. We abstracted and grouped qualitative findings relevant to fatigue adaptation in stroke survivors using constructs of the ALFCI as 4 main themes: 1) adaptive challenges, 2) adaptive work, 3) adaptive leadership and collaborative work, and 4) technical challenges and technical work. We found that stroke survivors encountered different aspects of challenges (e.g., physical dysfunction vs. mental distress) and utilized various adaptive work (e.g., conserving energy vs. restructuring normality) as well as what stroke survivors needed from healthcare professionals (e.g., basic knowledge about fatigue). The ALFCI provides a useful lens to synthesize qualitative findings regarding fatigue adaptation and therefore researchers can target different problems that need to be tackled for stroke survivors, care partners, or healthcare professionals, respectively.

